# Treatment of Gender in Research on Intervention Programs Targeting Social Isolation and Loneliness Among Older Adults: Scoping Review

**DOI:** 10.2196/72281

**Published:** 2026-02-18

**Authors:** Kenta Nomura, Naoto Kiguchi, Eisuke Inomata, Takeshi Nakamachi, Norikazu Kobayashi

**Affiliations:** 1 Graduate School of Rehabilitation Mejiro University Saitama Japan; 2 Department of Occupational Therapy Faculty of Health and Medical Sciences Ibaraki Prefectural University of Health Sciences Ibaraki Japan; 3 Department of Occupational Therapy Graduate School of Human Health Science Tokyo Metropolitan University Tokyo Japan; 4 Department of Psychology and Social Welfare Faculty of Psychology and Social Welfare Seigakuin University Saitama Japan

**Keywords:** social isolation, loneliness, intervention, program, gender, men, women, older, review, remote, in-person, scoping review

## Abstract

**Background:**

Social isolation and loneliness have considerable health implications. Research indicates that older men are generally more susceptible to social isolation compared with women, highlighting the need to integrate gender-responsive approaches in the development and implementation of interventions for mitigating social isolation and loneliness in later life.

**Objective:**

This study aimed to conduct a review of intervention programs targeting social isolation and loneliness, focusing on gender-specific considerations. Specifically, it aims to examine the gender composition (male-to-female ratio) of participants in intervention programs and identify and analyze intervention strategies that demonstrate gender-sensitive effectiveness.

**Methods:**

A scoping review was conducted as per the Joanna Briggs Institute manual for evidence synthesis. A comprehensive literature search, including hand searching, was conducted across 6 English-language databases, PubMed, MEDLINE, Cochrane, CINAHL, ScienceDirect, and Web of Science, for papers and reports published in 2013-2023. The authors, country, subjects, research design, intervention method, results, and mentions of gender for each included document were presented.

**Results:**

The study identified 1282 papers and reports, of which 10 were selected for analysis. Only 1 study reported a higher number of male participants compared with female ones; in contrast, all other studies included predominantly female samples. The studies assessed outcomes based on 2 indicators of social isolation, 4 indicators of loneliness, and 29 other indicators. Exercise and workshops proved effective for social isolation and loneliness, while meditation and laughter therapy were effective for loneliness. The intervention with the highest percentage of male participants (264/323, 82%) was a customized meditation program. Conversely, physical activities, social support, and community-based group health classes drew more female participants. In total, 8 studies did not mention gender in the discussion section, and none considered gender-specific issues in formulating research objectives and outcomes.

**Conclusions:**

Research on social isolation and loneliness has generally ignored the influence of gender. The review also indicated a gender bias in participant selection, with women markedly overrepresented in study samples. The study found that women tend to prefer interventions emphasizing conversations, shared experiences, and emotional exchange. In contrast, men showed the highest participation in a meditation program focused on self-dialogue, which required minimal interaction. Importantly, interventions aimed at promoting social interaction or participation are unlikely to succeed without consideration of gender-specific issues. Therefore, systematically identifying conditions necessary for effective interventions that target older men is crucial for guiding future research and program development.

**Trial Registration:**

Open Science Framework 10.17605/OSF.IO/83JQF; hhttps://osf.io/83jqf/overview

## Introduction

### Background

Social isolation is defined as “the objective lack or paucity of social contacts and interactions with family members, friends, or the wider community” [1]. Factors associated with social isolation include advanced age, male gender, presence of depressive symptoms, and low socioeconomic status [2]. The concept of social isolation is closely related to loneliness. According to Valtorta and Hanratty [1], loneliness is “a subjective negative feeling associated with a perceived lack of a wider social network (social loneliness) or the absence of a specific desired companion (emotional loneliness).” Scholars have defined social isolation as a lack of interaction with others and distinguished loneliness from subjective loneliness [3]. Although social isolation and loneliness are considered closely related, their definitions differ. Some studies have found only a weak correlation [4,5]; socially isolated individuals are not necessarily lonely, and vice versa. Despite these distinct definitions and realities, social isolation and loneliness are often studied together as overlapping concepts. Newall [6] also proposed that they should be examined in conjunction.

A growing body of evidence indicates that gender is a crucial factor influencing older adults’ experiences of social isolation and loneliness. For example, older men (ie, those aged 65 years and older) in Japan are more likely to experience social isolation compared with their female counterparts [7,8]. In a cross-sectional study, Nomura and Kobayashi [9] discovered that gender can influence the isolation-prevention strategies used by community-dwelling older people in Japan. The authors found that satisfaction with social activities (ie, the degree of satisfaction with activities involving participation in groups and organizations and interpersonal activities with others) and strategies for interactions helped older Japanese women maintain relationships with others. However, for older Japanese men, only satisfaction with social activities contributed to maintaining social relationships. Some studies also identify the need to focus intervention efforts on men [10]. Men are more likely to feel lonely compared with women for one reason: when confronted with challenges or distressing situations, women tend to respond emotionally and engage in conversation with others, whereas men tend to avoid stressors and neglect talking to others about them [11]. These findings underscore the importance of considering gender in the development of interventions targeting social isolation. Nevertheless, few intervention programs have appropriately accounted for gender. Furthermore, a comprehensive overview of findings on the salience of gender in social isolation research is lacking. Moreover, the treatment of gender in research on social isolation is inconsistent, given that researchers adopt different approaches to this issue.

Barreto et al [12] argued that research on loneliness has failed to address gender beyond the binary categories of male and female, thereby obscuring important influences. Other meta-analyses have also indicated a correlation between loneliness and gender. For example, Pollak et al [13] identified gender as a predictor and risk factor for loneliness and functional decline, while Haihambo et al [14] suggested that gender differences exist in both loneliness and social adaptability. However, these studies represent only one dimension of the analysis and fail to specifically focus on gender. Although gender is a critical determinant in understanding social isolation and loneliness, the prevalence of gender-focused interventions remains underexplored.

Several studies have examined intervention programs designed to address social isolation and loneliness. For example, Milligan [15] conducted a scoping review of Men’s Sheds and gender interventions to assess their impact on the health and well-being of older men. Men’s Sheds are community-based, mutual self-help groups established primarily in Australia and the United Kingdom, among other countries. These initiatives were intended to alleviate social isolation and loneliness among retired men by fostering purpose, companionship, and shared activities [16]. The review identified limited evidence supporting the impact of Men’s Sheds and other gender-focused social activities on the mental health and well-being of older men. However, this paper was published in March 2015; therefore, it does not cover research published after 2014. Fakoya et al [17] conducted a scoping review of studies on loneliness and social isolation interventions targeting older adults to identify effective interventions. The sample comprised 33 papers published until 2018. Findings indicated that the individual nature of experiences of social isolation and loneliness may impede the development and implementation of standardized interventions. The study concluded that no universal approach exists for addressing social isolation and loneliness, and interventions must be tailored to specific individuals and groups, as well as the severity of loneliness. Although existing studies encompass diverse interventions, a key limitation is that none explicitly considered gender as an influencing variable. Although a few studies address the potential impact of gender on interventions targeting the alleviation of social isolation and loneliness, the actual prevalence of such gender-focused interventions remains unclear.

### Objective

This study aims to comprehensively map intervention programs targeting social isolation and loneliness among older adults, with a particular emphasis on the integration of gender considerations in these programs. Specifically, it intends to (1) identify the male-to-female ratio of participants across studies and (2) determine effective intervention strategies that are responsive to gender differences. The results are expected to facilitate the development and implementation of new gender-focused intervention programs.

## Methods

### Research Design

A scoping review was conducted in accordance with the Joanna Briggs Institute (JBI) manual for evidence synthesis (hereafter, JBI framework) [18]. The JBI framework defines the purpose of a scoping review as identifying and analyzing knowledge gaps and examining how research is conducted within a given field [18]. The decision to conduct a scoping review was aimed at mapping the existing evidence and identifying areas that require further exploration rather than synthesizing findings, as customary in a systematic review. The review protocol was registered with the Center for Open Science Framework. No deviations were observed between the registered protocol and the content presented in this study. The inclusion criteria were based on the following definitions of participants, concept, context, and types of sources of evidence.

### Participants

The participants were older men and women residing in a community, regardless of whether they reported being socially isolated. The review also focused on older adults but did not strictly define the participants’ ages. Thus, age was excluded as an eligibility criterion given the diverse definitions of “elderly” across countries and studies.

### Concept

This study focused on the implementation of strategies for addressing social isolation and loneliness. However, studies examining the correlation of social isolation and loneliness with curfew restrictions during the COVID-19 pandemic were excluded. This study focused on cases of social isolation and loneliness in which underlying causes remained unclear and included gender as a variable. The present-day concept of gender is multifaceted; nevertheless, it is often reduced to a binary model that assigns specific biological and behavioral traits to men and women.

### Context

Social isolation and loneliness are influenced by various social factors, including geographic location, religious affiliation, and racial identity. This study does not impose any specific limitations regarding such factors; instead, it encompasses diverse community-based interventions.

### Types of Sources

This review covered various intervention studies, such as randomized controlled trials, nonrandomized controlled trials, and before-and-after studies. Quantifying the effectiveness of interventions targeting social isolation and loneliness is challenging. Therefore, the review also included websites, reports, and research papers while excluding observational studies, qualitative studies, reviews, and conference abstracts. The rationale for incorporating websites was twofold. First, a significant number of interventions that target social isolation and loneliness have not been disseminated through academic journals. Second, existing research on social isolation and loneliness has predominantly focused on academic publications, with nonacademic sources receiving minimal representation.

### Literature Search Strategy

The literature search strategy in this study was developed in consultation with a librarian at Mejiro University. A search was conducted on 6 databases (PubMed, MEDLINE, Cochrane, CINAHL, ScienceDirect, and Web of Science) on May 30, 2023, to identify relevant papers published within an 11-year period (2013-2023). The following terms were searched in combination: intervention, social isolation, loneliness, elderly, older adults, older people, older peoples, sex characteristics, sex, gender, and difference (Textbox 1). In addition to database searches, hand searches were conducted on the websites of 3 journals that frequently publish research on social isolation and loneliness: *Health and Social Care in the Community*, *Journal of Aging and Health*, and *Ageing & Society*. Furthermore, a hand search for gray literature was conducted on 2 websites, the Social Care Institute for Excellence and Connect2Affect, for the terms “social isolation” or “loneliness,” with the publication period limited to 2013-2023 (Multimedia Appendix 1).

**Search strategy.**

“(intervention)

and (((((social isolation)or(loneliness))

and (((aged)or(elderly))or((”“older people”“)or(”“older peoples”“))))

and((sex characteristics)or(((sex)or(gender))and(difference)))))”

### Literature Selection

Data extraction was performed by KN. All identified academic studies and relevant texts were uploaded to Rayyan [19]. Duplicate papers were omitted, followed by a screening. KN and EI conducted the initial and full-text screening of the literature, while NK was consulted in cases of disagreement. The sample included English-language papers featuring intervention programs for social isolation and loneliness. The following texts were excluded: conference abstracts; studies without mention of social isolation or loneliness in their objectives, methods, results, or discussion; and studies that focused on COVID-19 countermeasures.

### Data Analysis

The selected studies were presented in the form of a table that included the following information for each study: author, country of origin, participants, intervention method, outcome, and gender. An additional table was constructed to illustrate the male-to-female participant ratio per study and to classify the type of intervention (remote or in-person, group-based or individual, or a combination of these formats). Furthermore, the measures and outcome indicators used to evaluate the interventions were presented in tabular form. To enhance clarity and comparability, the primary outcome concepts were systematically categorized and organized according to their thematic relevance.

## Results

### Characteristics of the Selected Studies

**Figure 1:**
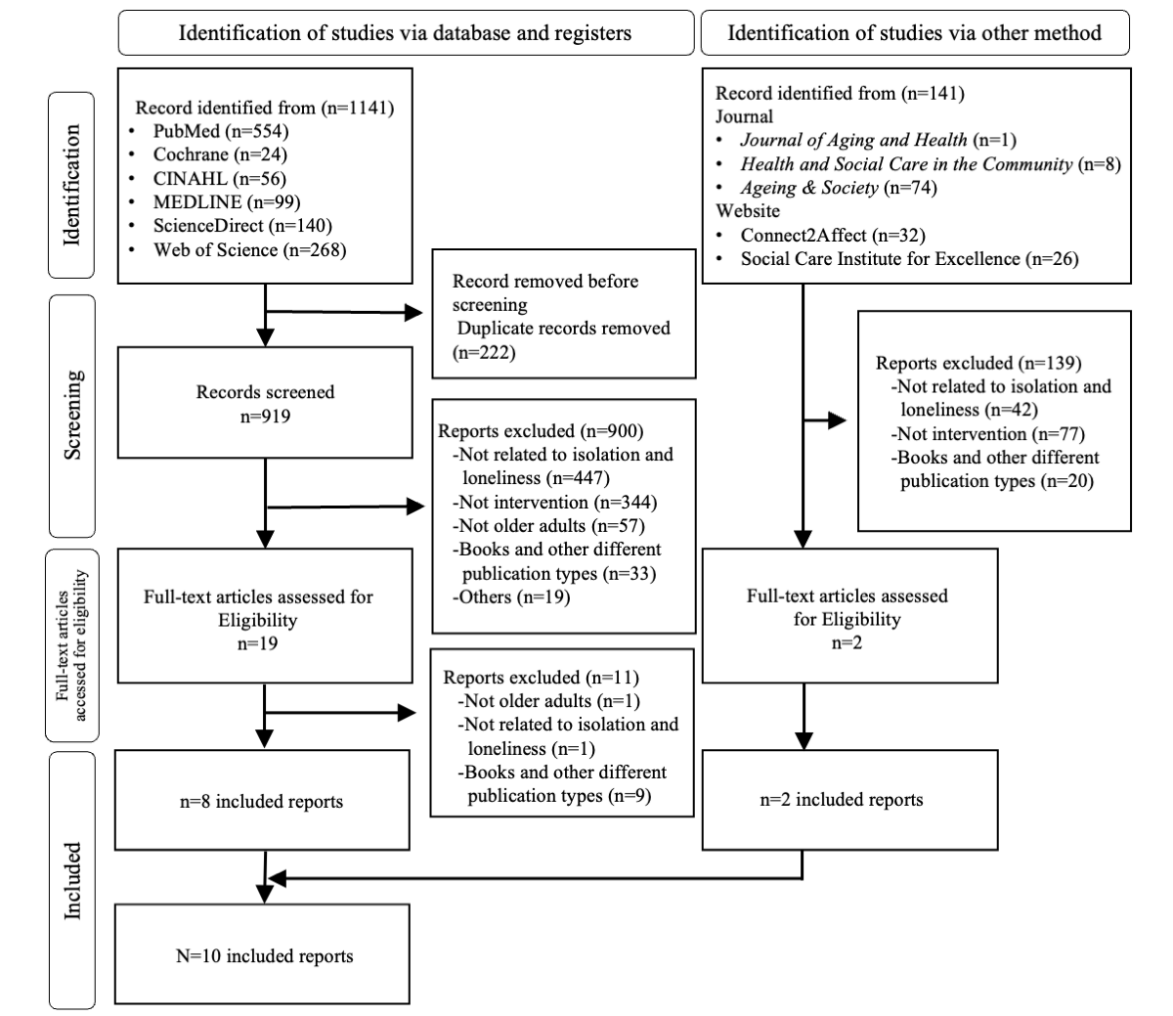
PRISMA (Preferred Reporting Items for Systematic Reviews and Meta-Analyses) flowchart of study selection.

The literature selection process was visualized as a flowchart in accordance with the PRISMA-ScR (Preferred Reporting Items for Systematic Reviews and Meta-Analyses extension for Scoping Reviews) checklist (Figure 1; Multimedia Appendix 2) [20]. The database search yielded 1141 studies, of which 222 were duplicates and were therefore excluded. The screening process yielded 19 studies, 8 [21-28] of which were included in the analysis. Of the 900 studies that were excluded during the screening process, 447 were unrelated to social isolation and loneliness, while 344 were not intervention studies. The hand search yielded 141 studies, of which 2 were included (Table 1). A total of 10 studies [21-30] were included in the final analysis. These studies described detailed interventions conducted in the United States (n=3 [23,27,28]), the United Kingdom (n=2 [29,30]), Singapore (n=1 [26]), Turkey (n=1 [25]), Spain (n=1 [21]), Ireland (n=1 [23]), and India (n=1 [22]; Table 1).

**Table 1:** Summary of included studies.

First author, date, and country	Participants (final analyzed individuals)	Intervention description	Result	Gender references
Ngiam et al, 2022 [26], Singapore	Older adults who resided in the southeast region of Singapore, aged >55 years, belonging to a lower socioeconomic status. 138 were included for analysis (59 male and 79 female).	Project Wire Up was a volunteer-led, one-on-one, goal-directed, and home-based digital literacy program: (1) equipped with smartphones and internet connection; (2) trained by volunteers for 6 sessions (1 to 2 hours per session) over 3 months that were held in the older adults’ homes; and (3) digitally connected to existing social networks.	There were significant improvements in digital literacy scores in the intervention group as compared to controls (mean difference 2.28, 95% CI 1.37-3.20; *P*<.001). There was no statistically significant difference in the University of California, Los Angeles 3-item loneliness scale, Lubben Social Network Scale-6, Personal Wellbeing Score, or EQ-5D Utility and visual analog scale score.	No mention
Santos-Olmo et al, 2022 [21], Spain	The sample (N=68: 18 male, 50 female) was 65 years of age or older; lived alone or lived with other people older than 65 years; had uncovered social and/or health needs; have little or no social support network; refused the assistance offered to cover their needs from the normalized social and/or health services.	The Psychological Support Service for Socially Isolated Elderly People (PSIE) facilitates contact between the elderly person and social and health services (both primary and specialized care) in their area, so that they can receive the care they need on each occasion.	Concerning the total score of the Health and Psychosocial Functioning (Total HoNOS65+, *t*=10.12, *P*<.001, Cohen *d*=1.49) and the Spanish adaptation of the Camberwell Needs Assessment Questionnaire for the Elderly (CANE; average number of unmet needs, *t*=19.99, *P*<.001, Cohen *d*=2.31), significant differences were also observed between pre- and posttreatment. Statistically significant changes were obtained in Global Assessment of Functioning (GAF; *t*=4.06, *P*<.001, Cohen *d*= 0.6) and the Spanish adaptation of the WHO Disability Assessment Short Scale (WHO-DAS-S; A. personal care and survival, *t*=8.82, *P*<.001, Cohen *d*=1.3).	The gender variable does not seem to have an influence on any of the outcome measures studied, except for alcohol use. PSIE has achieved positive results in both men and women, without gender appearing to be a relevant variable in these results.
Dodge et al, 2022 [27], United States	Participants with normal cognition or mild cognitive impairment were recruited from Portland, OR, and Detroit, MI. Key inclusion criteria included (1) age 75 years or older, (2) socially isolated. One hundred eighty-six participants (86 with normal cognition and 100 (52.8%) with mild cognitive impairment) were randomized into the experimental (n=94) or the control group (n=92).	The intervention group had video chats with trained study staff for 30 minutes per day, 4 times per week for 6 months (high dose), and then twice per week for an additional 6 months (maintenance dose). Both intervention and control groups received a phone call once per week (approximately 10 minutes duration) to assess changes in health and social activities.	After the induction period, the experimental group had higher global cognitive test scores (Montreal Cognitive Assessment [primary outcome]; 1.75 points [*P*=.03]). After induction, participants in the experimental group with normal cognition had higher language-based executive function (semantic fluency test [secondary outcome]; 2.56 points [*P*=.03]). At the end of the maintenance period, participants in the experimental group with mild cognitive impairment had higher encoding function (Craft Story immediate recall test [secondary outcome]; 2.19 points [*P*=.04]).	No mention
Pandya et al, 2021 [22], India	Intervention group older adults (IN_2_=166, 136 males, 30 females), the control group (CN_2_=157, 128 males, 29 females), who underwent no intervention	The key features of the meditation program were (1) postures interspersed with relaxation, (2) slowness in movements, and (3) inner watchful awareness.	There were significant mean differences in the posttest scores on loneliness, well-being, life satisfaction, and contentment outcomes of the intervention group, with high observed effect sizes (Cohen *d* range=2.43-8.78; *P*≤.01). The intervention group older adults reported that they were less lonely and experienced greater well-being, life satisfaction, and contentment posttest (ηp^2^ =0.71-0.78; *P*≤.01).	Men, middle class, married, and cohabitating participants, who also comprised a majority of the sample, were less lonely at the pretest phase as compared to women, upper class, single, and living alone. Gender was an important factor, and results showed that retired South Asian men were less lonely and more satisfied pretest as well as posttest.
Mays et al, 2021 [23], United States	Participants (n=382, 63 male, 315 female) were offered enrollment based on the following inclusion criteria: aged 50 years or older, community-dwelling, able to complete questionnaires, able to consent to participate in the study, and able to communicate in English.	Participants met with the program coordinator and selected from 1 of the 4 evidence-based programs: Tai Chi for Arthritis, Enhance Fitness, the Arthritis Foundation Exercise Program, and the Healthier Living Workshop. Group health class lasted 6 to 8 weeks.	Older adults who met with a health coach and participated in a single session of community health programs reported decreased loneliness (ER^a^ 0.931, 95% CI 0.895-0.968; *P*<.001) and social isolation (ER 1.033, 95% CI 1.016-1.050; *P*<.001) at 6 months post participation, compared to their baseline scores.	No mention
Zamir et al, 2020 [29], United Kingdom	Twenty-two residents aged ≥65 years (5 male, 17 female) across 3 British care homes	Twenty-two residents engaged with each other using “Skype quiz” sessions with the support of staff once a month over an 8-month trial. Residents met other residents from the 3 care homes to build new friendships and participate in a 30-minute quiz session facilitated by 8 staff members.	Analysis of the field notes revealed 5 themes of: residents with dementia remember faces, not technology, inter- and intraconnectedness, regaining sense of self and purpose, situational loneliness overcome, and organizational issues create barriers to long-term implementation.	No mention
Lawlor et al, 2019 [24], Ireland	Participants (n=40) from older (aged≥50 years) women’s groups from 4 different community centers	Intervention consisted of 3 face-to-face group education sessions, encouragement to enlist the support of a buddy (eg, spouse, partner, friend, or a group member), an information pack, and the option of weekly telephone contact. Each education session lasted approximately 20 minutes.	87% (40/46) of women consented to participate, and 78% (31/40) attended all education sessions. Few participants provided valid accelerometer data, but 63% (25/40) completed the HADS^b^ questionnaire at all time points. 85% of participants (34/40) were somewhat or very satisfied with their involvement in the study.	No mention
Alıcı et al, 2018 [25], Turkey	The study participants were older adults living in 2 nursing homes set up by foundations located in the capital of Turkey. Their ages were more than 65 years. A total of 50 older adults formed the intervention group (n=20; 9 males, 11 females) and control group (n=30; 14 males, 16 females).	Laughter therapy was conducted by the principal investigator 2 days a week, with one application during each session. The program involved performing yoga, breathing, and physical exercises, as well as laughter therapy. The program continued for 5 weeks for a total of 10 applications. The control group received no intervention.	A statistically significant difference (*P*<.001) between mean De Jong Gierveld Scale scores of the intervention (mean 7.15, SD 1.755) and control groups (mean 15.63, SD 5.027) was observed after the intervention. Median De Jong Gierveld Scale scores were significantly lower in the intervention group than in the control group. After therapy, the social loneliness score was significantly lower in the intervention group (mean 3.10, SD 1.553; *P*<.001) than in the control group (mean 6.90, SD 3.100). Post therapy, the emotional loneliness score was significantly lower (*P*<.001) in the intervention group (mean 4.05, SD 1.538) than in the control group (mean 8.73, SD 2.599).	No mention
Dodge et al, 2015 [28], United States	Eighty-three individuals participated (41 in the intervention group and 42 in the control group). Participants aged 70 years or older were included.	Daily 30-minute face-to-face communications were conducted during a 6-week trial period in the intervention group. The control group received only a weekly telephone interview.	Among the cognitively intact participants, the intervention group improved more than the control group on a semantic fluency test (*P*=.003) at the posttrial assessment and a phonemic fluency test (*P*=.004) at the 18-week assessments. Among those with mild cognitive impairment, a trend (*P*=.04) toward improved psychomotor speed was observed in the intervention group. No difference was found between intervention and control groups in the pre- to posttrial changes in the loneliness score, the secondary outcome.	No mention
Hind et al, 2014 [30], United Kingdom	The eligibility criteria included being aged ≥ 75 years, living independently, and having reasonable cognition. Fifty-six participants (control n=30, intervention n=26) were included in the intention-to-treat analysis group.	Manualized the telephone friendship (TF) with standardized training: (1) one-to-one befriending: 10- to 20-minute calls once per week for up to 6 weeks made by a volunteer befriender, followed by (2) TF groups of 6 participants: 1-hour teleconferences once per week for 12 weeks facilitated by the same volunteer. Control: usual health and social care provision.	The 2 groups were reasonably well matched with respect to baseline demographic characteristics. At 6 months post randomization, the SF-36 mental health mean (SD) scores were 77.5 (18.4) in the intervention group and 70.7 (21.2) in the control group, with a mean difference of 6.5 (95% CI –3.0 to 16.0); after adjusting for age, sex, and baseline scores, the mean difference was 9.5 (95% CI 4.5-14.5). In summary, over the 6-month follow-up period, there was no change in the SF-36 mental health scores in the intervention group, but there was a decline or deterioration in scores in the control group.	No mention

^a^ER: estimated ratio.

^b^HADS: Hospital Anxiety and Depression Scale.

The literature search was conducted using 2 complementary approaches: database and hand searches. Database searches were performed across the 6 abovementioned databases, yielding 1141 records, of which 8 studies [21-28] met the inclusion criteria. Hand searches were conducted across 3 journals and 2 websites, identifying 141 records, of which 2 [29,30] studies were eligible for inclusion.

In total, 10 studies [21-30] were included in the final analysis.

Table 1 presents a summary of the 10 papers included in the final analysis. All studies were published between 2013 and 2023. The United States and the United Kingdom were the countries with the highest representation in terms of intervention programs. Participants were generally older adults aged ≥50 years, with the majority being ≥65 years and predominantly female.

### Characteristics of Participants

**Figure 2:**
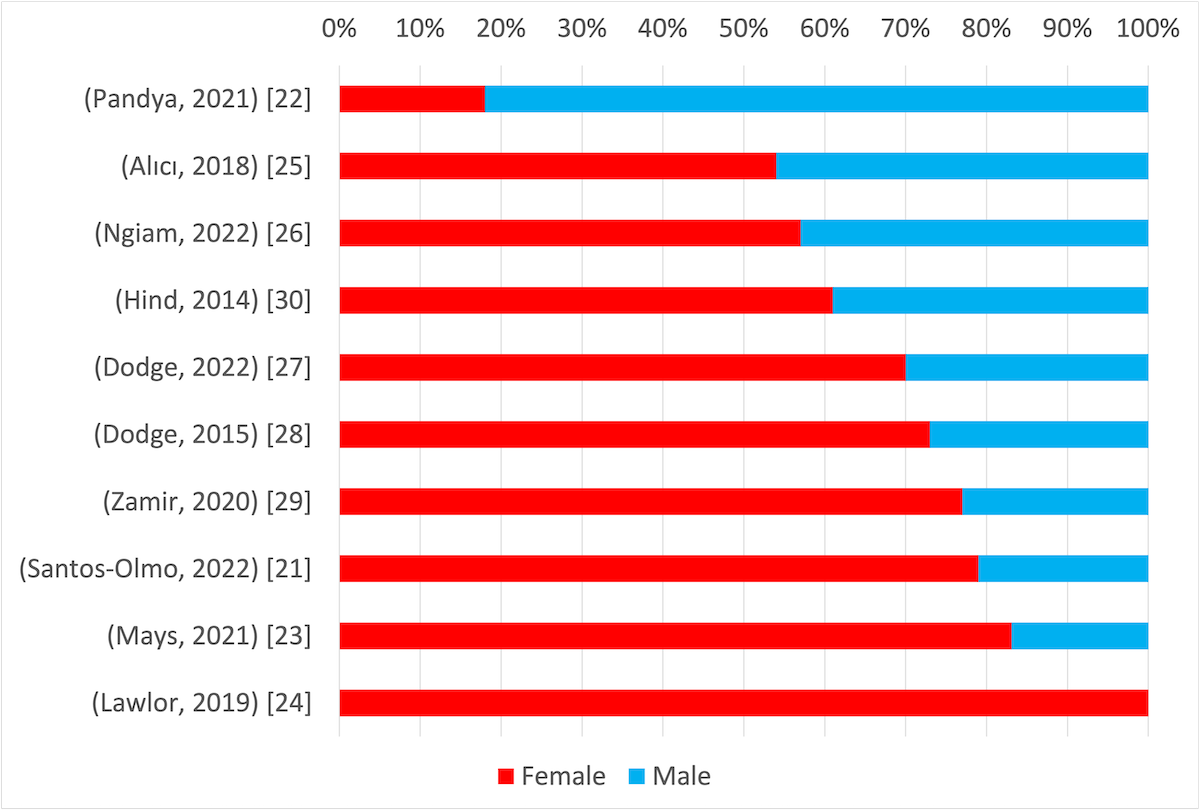
Gender ratio of participants [21-24, 26-30].

The 10 included studies [21-30] involved 1343 older adult participants, with sample sizes ranging from 22 to 382 (mean 134.3, SD 119.2; median 73, IQR 51.5-174). The proportion of female participants varied per study: approximately 10%-20%, 50%-60%, 60%-70%, 70%-80%, 80%-90%, and 100% women participated in 1 [26], 2 [21,27], 1 [22], 4 [23-25,29], 1 [28], and 1 [30] study, respectively (Figure 2 [21-30]). Overall, 8 documents incorporated the term “social isolation” or “loneliness” in their objectives, methods, or results. However, these documents did not explicitly address gender in the discussion section. In contrast, a few documents incorporated gender-related content in the discussion section. Studies referencing gender examined its impact on intervention effectiveness [21] and loneliness levels [22]. Among the included studies, the most common intervention method for those with the highest proportion of male participants (82%) was a personalized meditation program [22]. Conversely, studies involving women used different methods, including community-based group health classes [23] and physical activity and social support [24].

Only 1 [22] study reported a higher number of male participants compared with female ones; the remaining studies had a majority of female participants. One study [24] exclusively targeted older women, whereas no study focused solely on older men.

### Intervention Methods and Means

The studies used 4 types of study designs: randomized controlled trials (n=5), nonrandomized control trials (n=1), pre-post outcome studies (n=3), and action research (n=1). They assessed 5 types of in-group programs: a program intended to enhance access to social and health services [21], a meditation program [22], an exercise and workshop [23], a physical activity program [24], and a laughter therapy program [25]. Overall, 3 studies discussed individualized remote programs, including a digital literacy program [25], a video chatting program [27], and a face-to-face communication program [28]. Another study assessed a remote group program that used a Skype (Microsoft) quiz format [29]. Furthermore, 1 study evaluated a remote individual program and a remote group program based on telephone friendship [30] (Table 2). The exercise workshop [23] effectively alleviated social isolation and loneliness indicators; meanwhile, the meditation [22] and laughter therapy programs [25] effectively mitigated loneliness (Table 1).

The interventions were nearly evenly divided between “remote” (n=5) and “in-person” (n=5) formats. Notably, no individual in-person programs were reported; instead, group-based in-person interventions were the most common. In-person programs typically involved strategies such as facilitating access to social resources, meditation, exercise, and workshops. Remote programs included activities such as digital literacy training, video-based communication, and online conversational sessions.

**Table 2:** Intervention methods.

Remote		Face-to-face
**Individual**		
	Digital literacy program (Ngiam et al [26], 2022) Video chats (Dodge et al [27], 2022) Face-to-face communications (Dodge et al [28], 2015)	—^a^
**Group**		
	Skype quiz (Zamir et al [29], 2020)	Improving access to social and health services (Santos-Olmo et al [21], 2022) Meditation program (Pandya [22], 2021) Physical activity (Lawlor et al [24], 2019) Laughter therapy (Alıcı et al [25], 2018)
**Individual and group**		
	Telephone friendship (Hind et al [30], 2014)	Exercise and workshops (Mays et al [23], 2021)

^a^Not available.

### Measurement of Effectiveness

The 10 studies [21-30] assessed outcomes based on 2 indicators of social isolation, 4 indicators of loneliness, and 29 other indicators (Table 3). Among these, outcomes with positive effects were observed for the Duke Social Support Index [23] for social isolation, the 3-item loneliness scale of the University of California, Los Angeles [23], the De Jong Gierveld Loneliness Scale [25], and the 6-item De Jong Gierveld Loneliness Scale [22].

The selected studies presented relatively few measures for assessing social isolation and loneliness; however, they used a broad range of indicators to evaluate cognitive function, well-being, satisfaction, and other psychosocial domains. For outcomes related to social isolation or loneliness and cognitive function, the number of studies that reported significant effects was approximately equal to those reporting no effects.

**Table 3:** Outcomes and effectiveness.

Categories and outcomes				Effective		Ineffective
**Social isolation**						
	LSNS-6^a^			—^b^		Ngiam et al [26], 2022
	DSSI^c^			Mays et al [23], 2021		—
**Loneliness**						
	UCLA-3^d^			Mays et al [23], 2021		Ngiam et al [26], 2022
	The De Jong Gierveld Loneliness Scale score			Alıcı et al [25], 2018		—
	3-item loneliness scale			—		Dodge et al [27], 2015
	DJGLS-6^e^			Pandya et al [22], 2021		—
**Other**						
	**Cognition**					
		Trail making test	—		Dodge et al [28], 2015	
		Mini mental state examination	—		Dodge et al [28], 2015	
		Cogstate computerized tests	—		Dodge et al [28], 2015	
		The Stroop test	—		Dodge et al [28], 2015	
		MoCA^f^	Dodge et al [27], 2022		—	
		Craft Story immediate recall test	Dodge et al [27], 2022		—	
		The Consortium to Establish a Registry for Alzheimer Disease word list learning	—		Dodge et al [28], 2015	
		The Consortium to Establish a Registry for Alzheimer Disease word list delayed recall	—		Dodge et al [28], 2015	
		CAMCI^g^	—		Dodge et al [28], 2015	
	**Fluency**					
		Semantic fluency test	Dodge et al [27], 2022) Lawlor et al [24], 2019)		—	
		The composite of verbal fluency for letters (F, A, and S)	Dodge et al [28], 2015)		—	
		Verbal fluency for the category animals	Dodge et al [28], 2015)		—	
	**Qualitative method**					
		WHO-DAS-S^h^	Santos-Olmo et al [21], 2022		—	
		Thematic analysis	Zamir et al [29], 2020		—	
		framework analysis	Lawlor et al [24], 2019)		—	
	**Well-being**					
		PWS^i^	—		Ngiam et al [26], 2022	
		WEMWBS^j^	Pandya et al [22], 2021		—	
	**QoL^k^**					
		EQ-5D	—		Ngiam et al [26], 2022	
		SF-36^l^	Hind et al [30], 2014		—	
	**Psychosocial functioning**					
		HoNOS65+^m^	Santos-Olmo et al [21], 2022)		—	
		GAF^n^	Santos-Olmo et al [21], 2022)		—	
	**Satisfaction**					
		SWLS^o^	Pandya et al [22], 2021		—	
		CLAS^p^	Pandya et al [22], 2021		—	
	**Emotion**					
		TDAS^q^	Alıcı et al [25], 2018		—	
		NIHTB-EB^r^	Dodge et al [27], 2022		—	
	**Digital literacy**					
		Digital literacy score	Ngiam et al [26], 2022		—	
	**Needs**					
		CANE^s^	Santos-Olmo et al [21], 2022		—	
	**Depression**					
		Hospital Anxiety and Depression Scale	—		Lawlor et al [24], 2019	
	**fMRI^t^**					
		fMRI analyses	Dodge et al [27], 2022		—	

^a^LSNS-6: Lubben Social Network Scale-6.

^b^Not available.

^c^DSSI: The Duke Social Support Index.

^d^UCLA-3: University of California, Los Angeles 3-item loneliness scale.

^e^DJGLS-6: The De Jong Gierveld Loneliness Scale (six-items).

^f^MoCA: Montreal Cognitive Assessment.

^g^CAMCI: Computer assessment of mild cognitive impairment.

^h^WHO-DAS-S: World Health Organization Brief Disability Assessment Scale.

^i^PWS: Personal Well-being Score.

^j^WEMWBS: Warwick Edinburgh Mental Well-being Scale.

^k^QoL: quality of life.

^l^SF-36: 36-Item Short Form Health Survey.

^m^HoNOS65+: Home Health Outcome Scales for People Over 65.

^n^GAF: Global Assessment of Functioning.

^o^SWLS: The Satisfaction with Life Scale.

^p^CLAS: The Contentment with Life Assessment Scale.

^q^TDAS: The Turkish Death Anxiety Scale.

^r^NIHTB-EB: National Institutes of Health-toolbox emotional battery.

^s^CANE: Camberwell Needs Assessment Questionnaire for the Elderly.

^t^fMRI: functional magnetic resonance imaging.

## Discussion

### Principal Findings

In this review, a total of 1282 papers were identified, of which only 10 [21-30] were eligible for analysis. Studies were excluded primarily because they did not specifically examine social isolation or loneliness or were merely cross-sectional investigations that emphasized the need for interventions. Of the included studies, East Asia was represented only by Singapore; notably, no research was included from Japan despite its status as a super-aged society.

Regarding the interventions, the methods were evenly distributed between remote and in-person methods. In total, 5 [26-30] of the 10 (50%) studies [21-30] used remote interventions (eg, digital literacy and video chats), while the other 5 studies [21-25] used in-person interventions (eg, exercise, meditation, and laughter therapy). However, none of them combined remote and in-person approaches.

A significant gender bias was observed; in 9 studies [21-24,26-30], most participants were women. Among studies in which women accounted for ≥80% or more of the participants, interventions frequently emphasized communication, shared emotional expression, and group-based activities. Conversely, the study with the highest proportion of male participants (82%) featured a meditation program characterized by self-dialogue and individual reflection.

Importantly, only 2 [21,22] of the 10 studies [21-30] discussed the impact of gender on intervention outcomes, whereas no study focused on gender-specific issues as a primary research objective. Furthermore, the use of specific indicators for social isolation and loneliness was limited; indicators were typically substituted by measures of cognitive function or quality of life, depending on the study’s specific objectives.

### Comparison With Prior Work

The finding that 50% of the studies used remote interventions represents a significant increase compared with the result of a previous scoping review conducted by Fakoya et al [17]. The authors found that only 15% (5 of 33) of interventions cited in papers published between 1984 and 2018 were remote. This trend aligns with the growing efficacy and development of remote tools [31,32], likely accelerated by the global COVID-19 pandemic. However, while Sen et al [32] recommended the use of online and offline resources to enhance efficacy, this review found no studies adopting such a hybrid approach.

The predominance of female participants is consistent with previous literature emphasizing that research on social isolation and loneliness is biased toward women [33,34]. This finding contradicts evidence suggesting that older men are more prone to social isolation compared with women [7,8].

Regarding intervention preferences, the findings support existing theories on gender differences. Female participants’ preference for exchanging experiences in supportive settings mirrors the findings of Venter et al [35]. In contrast, the male preference for the meditation program aligns with research proposing that men gravitate toward task-oriented activities, autonomy, and less intimate social networks, as seeking help or emotional disclosure can be perceived as vulnerability [36].

The lack of gender-specific analysis in the included studies is in contrast with the recommendations of Santos-Olmo et al [21] and Wen et al [10], who argued that future research needs to prioritize gender as a variable for predicting the effectiveness of interventions. The absence of studies conducted in Japan is also a concern, given the cultural specificity of isolation [13,37], thus highlighting a gap between Japan’s urgent policy needs [38] and the available evidence base.

### Strengths and Limitations

#### Strengths

The primary strength of this study is its explicit focus on gender. To the best of our knowledge, this is the first literature review on social isolation and loneliness that considers gender as a central variable. Additionally, relevant studies were identified using a rigorous methodology based on multidisciplinary databases.

#### Limitations

First, the review may not have captured all relevant studies, partially because the study period overlapped with the COVID-19 pandemic, potentially causing delays in publication. Second, the literature search concluded in May 2023, excluding subsequent studies. Third, despite manual searches, some high-quality sources or community-based practices that indirectly address isolation (eg, neighborhood association activities) may have been missed. Finally, as a scoping review, no quality assessment of the included papers was conducted; thus, the quality of evidence may vary.

### Future Directions

Gender-specific interventions: a clear need exists to develop and implement new gender-focused intervention programs.

For older women: interventions should prioritize group-based activities that foster dialogue, mutual understanding, and emotional connection.

For older men, future efforts must rigorously identify criteria for interventions targeting men. Programs should incorporate activities that enable self-expression without overt emotional disclosure, such as those emphasizing skill use, personal autonomy, and a sense of contribution.

Research design: future studies must move beyond merely reporting gender demographics. Studies should include gender as the primary variable in order to analyze its impact on intervention outcomes. A reexamination of previous studies that failed to show significant effects could reveal whether the lack of gender consideration was a contributing factor.

Hybrid approaches: given the increased popularity of remote interventions and the established value of in-person interaction, future interventions should aim to combine remote and in-person methods. Leveraging the experience of online-based interventions to create hybrid models could enhance the precision and efficacy of countermeasures against social isolation and loneliness.

### Conclusions

This scoping review, which aimed to examine the composition of participants by gender and gender-sensitive strategies, confirms that research on social isolation generally underestimates gender. This finding reveals a bias in which women are markedly overrepresented in study samples, contradicting evidence that older men are more susceptible to isolation. Critically, no study considered gender-specific issues in its objectives, undermining the efficacy of general interventions. We found gender-based differences in preferences: women favored conversation and emotional exchange, while men exhibited the highest proportion of participation in self-dialogue meditation programs. Therefore, a clear need exists to develop new gender-focused intervention programs and systematically identify conditions for effective interventions that specifically target older men.

## Appendix

Multimedia Appendix 1 Search strategy.

Multimedia Appendix 2 PRISMA-ScR checklist.

## Abbreviations

JBI: Joanna Briggs Institute
PRISMA-ScR: Preferred Reporting Items for Systematic Reviews and Meta-Analyses extension for Scoping Reviews

## Data Availability

All data generated or analyzed during this study are included in this published paper.
