# Supplementary material for: Treatment of Gender in Research on Intervention Programs Targeting Social Isolation and Loneliness Among Older Adults: Scoping Review
Source: Interact J Med Res. 2026 Feb 18;15:e72281. doi: 10.2196/72281 (PMC12961394; doi:10.2196/72281)
Supplement: Multimedia Appendix 1 [file ijmr_v15i1e72281_app1.docx]

Search Strategy

Treatment of Gender in Research on Intervention Programs Targeting Social Isolation and Loneliness: A Scoping Review

|  |  |
| --- | --- |
| PubMed | "(intervention  AND ((((("social isolation") or(loneliness))  AND (((aged) or(elderly)) "or((""older people""") "or(""older peoples""")  AND (("sex characteristics") or(((sex) or(gender and(difference "  Filter: 2013-2023 |
| MEDLINE | "(intervention  AND ((((("social isolation") or(loneliness))  AND (((aged) or(elderly)) "or((""older people""") "or(""older peoples""")  AND (("sex characteristics") or(((sex) or(gender and(difference "  Filter: 2013-2023 |
| Cochrane | "(intervention  AND ((((("social isolation") or(loneliness))  AND (((aged) or(elderly)) "or((""older people""") "or(""older peoples""")  AND (("sex characteristics") or(((sex) or(gender and(difference "  Filter: 2013-2023 |
| CINAHL | "(intervention  AND ((((("social isolation") or(loneliness))  AND (((aged) or(elderly)) "or((""older people""") "or(""older peoples""")  AND (("sex characteristics") or(((sex) or(gender and(difference "  Filter: 2013-2023 |
| ScienceDirect | "(intervention  AND ((((("social isolation") or(loneliness))  AND (((aged) or(elderly)) "or((""older people""") "or(""older peoples""")  AND (("sex characteristics") or(((sex) or(gender and(difference "  Filter: 2013-2023 |
| Web of Science | "(intervention  AND ((((("social isolation") or(loneliness))  AND (((aged) or(elderly)) "or((""older people""") "or(""older peoples""")  AND (("sex characteristics") or(((sex) or(gender and(difference "  Filter: 2013-2023 |
